# IL-6 Inhibition Partially Ameliorates Maternal Immune Activation-Induced Autism-Like Behavioral Abnormalities in Mice

**DOI:** 10.3390/cimb47100852

**Published:** 2025-10-16

**Authors:** Xiaoyun Zhang, Weili Luo, Kaiyue He, Yuping Li, Yan Chen, Zhipeng Xu, Zi-Kai Zhou

**Affiliations:** 1 Zhongshan Institute for Drug Discovery, Shanghai Institute of Materia Medica, Chinese Academy of Sciences, Zhongshan 528400, China; zhangxiaoyun1@zidd.ac.cn (X.Z.); liyuping@zidd.ac.cn (Y.L.); 2 State Key Laboratory of Discovery and Utilization of Functional Components in Traditional Chinese Medicine, School of Pharmaceutical Sciences, Guizhou Medical University, Guiyang 561113, China; luoweili521@zidd.ac.cn (W.L.); s0710189@sina.com (Y.C.); 3 State Key Laboratory of Reproductive Medicine and Offspring Health, Department of Pathogen Biology and Immunology, Nanjing Medical University, Nanjing 211166, China; hekaiyue0307@163.com

**Keywords:** maternal immune activation, autism spectrum disorder, interleukin-6, behavioral tests

## Abstract

Prenatal maternal immune activation (MIA) has been implicated in autism spectrum disorder (ASD) pathogenesis, with interleukin-6 (IL-6) identified as a key inflammatory mediator. We investigated the therapeutic potential of IL-6 inhibition in an MIA mouse model induced by *Toxoplasma gondii* soluble tachyzoite antigen (STAg). Adult MIA offspring received systemic administration of the IL-6-neutralizing antibody (MP5-20F3) or isotype control, followed by behavioral assessments one week later. Open field and elevated plus maze tests revealed heightened anxiety-like behaviors in the STAg offspring, which were largely reversed by IL-6 inhibition. Reciprocal social interaction tests showed diminished sociability in the STAg offspring, which was partially restored by IL-6 inhibition. However, core ASD-like features, including impaired social preference and recognition in the three-chamber test, as well as increased repetitive behaviors, remained resistant to IL-6 inhibition. These findings demonstrate that STAg-induced MIA elicits anxiety-like and ASD-like phenotypes in adult offspring, with IL-6 playing an important role in anxiety-like behaviors and social interaction deficits. Systemic IL-6 inhibition partially ameliorates behavioral abnormalities. This study suggests that IL-6-targeted therapies may address a subset of ASD-related symptoms, and comprehensive strategies are needed for broader efficacy.

## 1. Introduction

According to epidemiological findings, pregnancy-related maternal infection and the consequent maternal immune activation (MIA) have been implicated in the pathogenesis of autism spectrum disorder (ASD) in offspring [1,2,3]. Infections caused by diverse pathogens, including bacteria, viruses, and parasites, are the primary contributing factors to MIA [4,5,6,7]. During the critical period of rapid embryonic brain development, MIA can mediate a series of pathological processes, including sustained inflammatory cascades [8,9], glial cell dysfunction [10,11], defects in synapse formation and pruning [12,13], neurotransmitter imbalances, and neural circuit defects [14,15]. These long-term consequences result in offspring exhibiting autism-like phenotypes in adulthood, such as social interaction impairments, recurrent stereotypical behaviors, limited interests, and sensory processing abnormalities [16,17,18,19].

Our previous study utilized *Toxoplasma gondii* soluble tachyzoite antigen (STAg) to trigger MIA in mice, establishing the first animal model of MIA using parasite antigens. *T. gondii* [6,7], an opportunistic apicomplexan protozoan and obligate intracellular parasite, infects approximately two billion people, or one-third of the global population [20,21]. Unlike the bacterial mimetic lipopolysaccharide (LPS) or the viral mimetic poly(I:C)—chemical substances commonly used to induce MIA in animal models—these approaches directly activate the innate immune system but do not reproduce the full spectrum of immune responses normally induced by infectious pathogens, particularly pathogen-specific adaptive cellular immune reactions mediated by T cells. In contrast, STAg comprises complex biomacromolecules, including proteins, lipids, glycoproteins, and others [22,23,24], and therefore activates not only innate immune responses but also pathogen-specific adaptive cellular immune reactions, such as T cell immunity, thereby representing a broader spectrum of pathological conditions [7,25]. Consistent with this, our previous work showed that STAg administration in pregnant dams induced a canonical pro-inflammatory T cell profile characterized by elevated T helper 1 (TH1) and T helper 17 (TH17) cells and reduced regulatory T (Treg) cells, thus validating the maternal inflammatory state [7].

At the cellular level, CD4+ T cells in the offspring mice from dams exposed to STAg-induced MIA exhibited a pro-inflammatory profile. At the molecular level, peripheral blood and tissue lysates from the whole brain, hippocampus, striatum, and cortex exhibit markedly elevated expression of the pro-inflammatory cytokine IL-6 [7]. Given the profound immunosuppressive functions of regulatory T cells (Tregs), our previous study investigated the effects of adoptive Treg cell transfer in adult MIA offspring mice at 8 weeks of age. One week after Treg transfer (5 × 10^5^ cells per mouse, i.v.), we observed that Treg cells largely reversed the CD4+ T cell pro-inflammatory profile, reduced serum IL-6 levels, decreased the number of pro-inflammatory astrocytes, and rescued ASD-related social behaviors [7], suggesting significant therapeutic potential for Treg cell therapy in ASD.

However, Treg cells constitute only 2–5% of lymphocytes and are difficult to isolate in large quantities, limiting their clinical applicability. IL-6, a key mediator of neuroinflammation, has been found to be upregulated in ASD patients and may alter brain connectivity in both humans and mice [26,27,28,29]. These findings suggest that IL-6 contributes to aspects of ASD pathogenesis. Therefore, investigating whether neutralizing IL-6 with antibodies can alleviate autism-like behaviors in the STAg-MIA mouse model presents an important question.

## 2. Materials and Methods

### 2.1. Mice

Wild-type C57BL/6 mice were housed and bred under standardized conditions in the Animal Laboratory Resource Facility at the Zhongshan Institute for Drug Discovery and Nanjing Medical University. A controlled environment was maintained for the mice, with a 12-h light/dark cycle, 22 °C temperature, and 50% humidity. All experimental procedures involving animals strictly adhered to the Regulations for the Administration of Affairs Concerning Experimental Animals (enacted on 1 November 1988). The experimental protocols were reviewed and approved by both the Institutional Animal Care and Use Committees of Zhongshan Institute for Drug Discovery (approval number: 2024-02-ZZK-02, approved on 1 March 2024) and Nanjing Medical University (approval number: 101025-1, approved on 4 March 2021).

### 2.2. Preparation of Soluble Tachyzoite Antigen (STAg)

The RH strain tachyzoites were grown in HeLa cells. A total of 3 × 10^6^ cells were incubated with the same number of tachyzoites at 37 °C for 72 h and at 25 °C for 120 h. When nearly 80% of the host cells were destroyed, the tachyzoites were harvested and counted to establish new infections. The parasites were passed through a nucleopore membrane of 3 μm supplied by Shanghai GUCH Nuclear Pore Membrane Technology Co., Ltd. (Shanghai, China). and then washed twice with PBS. Following centrifugation, the pellet was used for additional analysis. The protein concentration of STAg was determined using Bio-Rad’s bicinchoninic acid (BCA) protein assay kit (Richmond, CA, USA).

### 2.3. STAg-Induced Maternal Immune Activation

Following overnight mating, E0.5 was determined as the day when a vaginal plug was observed. At E14.5, pregnant females were randomly assigned to receive a single intraperitoneal injection of either PBS or 90 μg STAg. Each dam received an injection, was put back in its cage, and was not disturbed until delivery. The mice were housed in groups of up to five individuals per cage with their same-sex littermates after the offspring were weaned on postnatal day 21 (P21).

### 2.4. IL-6 Inhibition

Offspring derived from dams treated with either PBS or STAg were randomly assigned within each maternal treatment group to receive 100 µg of either an isotype control antibody (Bio X Cell, #BE0088, RRID: AB_1107775) or an anti-mouse IL-6-neutralizing antibody (clone MP5-20F3, Bio X Cell, #BE0046, RRID: AB_1107709) via tail vein injection for in vivo IL-6 neutralization, resulting in four experimental groups (PBS + IgG, STAg + IgG, PBS + MP5, and STAg + MP5).

### 2.5. Behavioral Tests

In this study, behavioral assessments were conducted exclusively on male offspring to avoid potential sex-related variability. The experiments were conducted using previously developed protocols, with minor adjustments detailed below. The mice were assessed at 9–10 weeks of age and transferred to the testing area 24 h before testing to allow acclimation. Prior to each mouse’s examination, all testing equipment was thoroughly cleaned with 75% ethanol to reduce smell influence. All behavioral tests were conducted in the light phase, and Noldus Ethovision XT software (version 11.5, Noldus, Wageningen, The Netherlands) was used to record and blindly assess the animals’ behaviors. To minimize litter effects, offspring were randomly selected from each dam, with 3–5 mice per litter included, depending on group size. All behavioral assessments were performed by investigators blinded to the group allocation.

#### 2.5.1. Open Field Test

For 10 min, each mouse was left alone in a 50 × 50 × 50 cm opaque plastic chamber to explore at will. A central (36 cm in diameter) and a peripheral zone were created within the open field arena.

#### 2.5.2. Three-Chamber Sociability Test

The social behavior test was performed in a three-chambered apparatus (20 × 40 × 22 cm) connected by doors (12 × 6 × 0.5 cm) allowing free movement. During a 10-minute habituation, the subject mouse explored the arena with empty wire cages in the side chambers, and any mice showing strong innate side preference were excluded. Following habituation, the mouse spent 10 min exploring each chamber before being placed in one side chamber with an unknown age-matched C57BL/6J male (Stranger 1). In the next phase, the mouse was kept in the central chamber for 10 min, while a second novel mouse (Stranger 2) was introduced to the previously empty cage, after which the test mouse explored all chambers for another 10 min, with stranger locations randomly assigned. Sniffing time was manually recorded; % [(time sniffing Stranger 1 − time sniffing Empty)/(time sniffing Stranger 1 + time sniffing Empty)] and % [(time sniffing Stranger 2 − time sniffing Stranger 1)/(time sniffing Stranger 1 + time sniffing Stranger 2)] were used to calculate the social approach and social novelty preferences, respectively.

#### 2.5.3. Marble Burying Behavior

Each mouse was housed individually in a clean, transparent cage (36 × 20 × 13 cm) containing a 5 cm layer of bedding and 15 glass marbles (15 mm in diameter) arranged in a 3 × 5 grid on the floor. After a 30 min testing period, the mice were removed, and the number of buried marbles—defined as those at least two-thirds covered by bedding—was recorded.

#### 2.5.4. Elevated Plus Maze Test

The elevated plus maze was made of white plastic, with two open arms (35 × 5 cm) and two enclosed arms (identical dimensions but with 15 cm high walls). The apparatus was elevated 60 cm above ground level. The mice were individually placed in the central platform, facing an open arm, and allowed to freely explore for 10 min.

#### 2.5.5. Reciprocal Social Interaction Test

The test mouse was first habituated to an empty plastic cage for 10 min before social testing. In the age-matched peer assay, an unfamiliar male C57BL/6J mouse (age- and weight-matched, tail-marked with red dye) was introduced for a 10-minute free interaction session, while the juvenile target assay used an unfamiliar juvenile male instead. All sessions were video-recorded using Noldus Ethovision XT, with social interaction time (including nose-to-nose/anogenital sniffing, following, chasing, mounting, grooming, and crawling over/under) scored by a treatment-blinded observer using consistent criteria across both assays.

#### 2.5.6. Self-Grooming

The experimental mouse was first acclimated for 5 min in a novel cage containing fresh bedding before behavioral recording commenced. Video tracking was performed continuously for 10 min using the Noldus Ethovision XT. A trained observer, unaware of treatment group assignments, quantified the total duration of grooming behaviors (including facial, head, and body scratching/rubbing) through manual scoring with a stopwatch.

#### 2.5.7. Nest Building Assay

Nest-building behavior was evaluated by housing individual test mice in fresh cages, each provisioned with one sterile 5 × 5 cm cotton square. After 24 h, nests were photographed and scored based on material utilization and structural organization using a 5-point scale: 1, minimal disturbance (>90% intact material); 2, partial processing (50–90% remaining); 3, shredded material without nest formation; 4, flattened nest structure; or 5, complete nest with defined walls.

### 2.6. ELISA

The manufacturer’s instructions for serum preparation and IL-6 protein assays were followed (RayBiotech, Peachtree Corners, GA, USA, ELM-IL6-1). Blood samples were centrifuged after being left to coagulate at room temperature, and the resulting serum was stored at −80 °C. Brain tissues were collected promptly, samples were kept at −80 °C, and rapid liquid nitrogen freezing was carried out. Brain tissue was homogenized in lysis solution containing protease inhibitors in order to prepare the lysate, and a BCA protein assay kit was used to measure the protein contents (Thermo Fisher Scientific, Waltham, MA, USA). IL-6 levels were determined by interpolating from a standard curve, and ELISA experiments were carried out precisely in accordance with the manufacturer’s procedure. All sample processing and ELISA measurements were conducted by investigators blinded to group allocation.

### 2.7. Statistical Analyses

SPSS version 19.0 was used for all statistical analyses. The data are presented as mean ± standard error of the mean (SEM). Student’s *t*-test was used to evaluate the differences between the two groups. One-way analysis of variance (ANOVA) and Bonferroni post hoc tests were used for multiple group comparisons. All data analyses were performed by investigators blinded to group allocation to ensure objectivity. Statistical significance was set at *p* < 0.05. The following symbols indicate significance levels: * *p* < 0.05, ** *p* < 0.01, *** *p* < 0.001, and **** *p* < 0.0001.

## 3. Results

**Figure 1:**
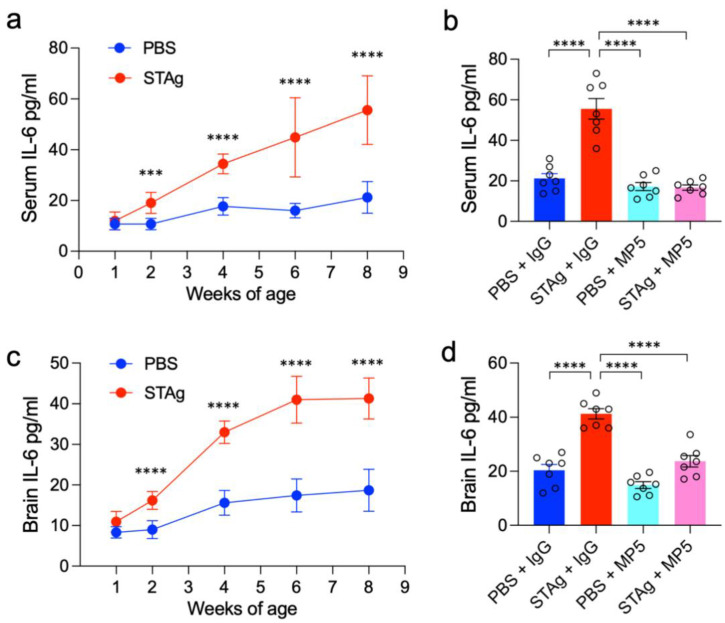
Postnatal IL-6 protein levels and efficacy of IL-6 inhibition in serum and brain. (**a**,**c**) ELISA results illustrating the gradual increase in IL-6 protein levels in (**a**) serum and (**c**) whole-brain lysate samples during postnatal development in offspring. The data are shown as mean ± s.e.m. Student’s *t*-test. *n* = 7 mice from 3 independent dams per group. (**b**,**d**) ELISA results showing the effective reduction of IL-6 protein levels in (**b**) serum and (**d**) whole-brain lysate after systemic treatment with the MP5 antibody (IL-6-blocking antibody) at 8 weeks of age. An isotype-matched IgG served as a control for the antibody treatment. The data are shown as mean ± s.e.m. *n* = 7 mice from 3 independent dams per group; statistical analysis was performed using one-way ANOVA with Bonferroni post hoc test; *** *p* < 0.001, **** *p* < 0.0001. All statistical information, group averages, and specific test results are presented in Extended Data Table S1.

**Figure 2:**
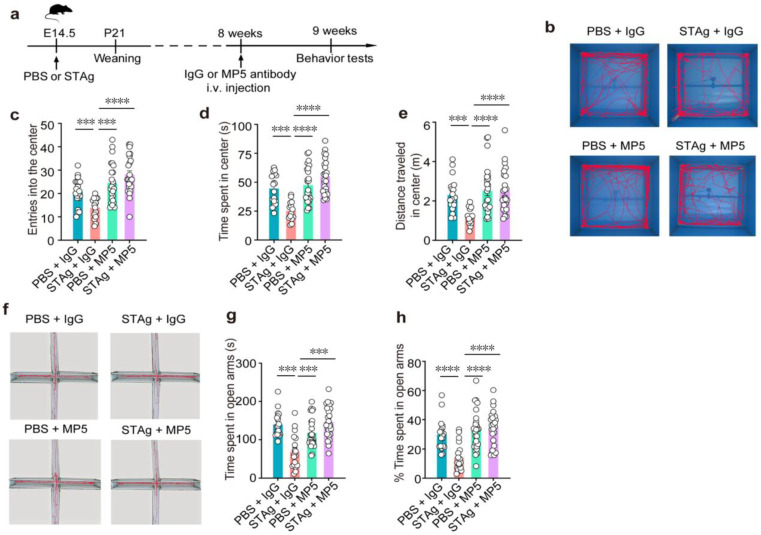
IL-6-neutralizing antibody MP5 alleviates MIA-induced anxiety-like behavior. (**a**) Schematic diagram of the experimental design. PBS or STAg offspring were i.v. injection with blocking antibody against IL-6 (MP5) or isotype-control antibody (IgG) at 8 weeks. After one week, behavior tests were performed. (**b**–**e**) Open fields test. (**b**) Representative open field test of mouse movement samples. Total number of entries (**c**) into, total time spent (**d**), and distance traveled (**e**) in the center (*n* = 20, PBS + IgG, *n* = 19, STAg + IgG; *n* = 20, PBS + MP5; *n* = 19, STAg + MP5; mice from 5 independent dams per group). (f–h) Elevated plus maze test. (**f**) Sample routes in the elevated plus maze test that demonstrate movement. Total time (**g**) and percentage of time (**h**) spent in the open arms (*n* = 19, PBS + IgG, *n* = 18, STAg + IgG; *n* = 19, PBS + MP5; *n* = 18, STAg + MP5; mice from 5 independent dams per group). Data are presented as mean  ±  s.e.m. Panels (**c**–**h**): One-way ANOVA with Bonferroni post hoc test. *** *p* < 0.001, **** *p* < 0.0001. All statistical information, including sample sizes, group averages, and specific test results, is presented in Extended Data Table S1, and a summary of behavioral outcomes across all tests is provided in Supplementary Table S2.

The IL-6 receptor antibody Tocilizumab (TCZ) has been clinically used to treat a wide range of diseases. Here, we used a monoclonal antibody (MP5-20F3) that recognizes and neutralizes IL-6 in mice to inhibit IL-6 signaling cascades [30]. As previously documented, we administered 90 µg STAg or vehicle (PBS) intraperitoneally (i.p.) to pregnant C57BL/6 mother mice on E14.5 in order to induce STAg-MIA [7]. IL-6 protein levels in both serum and whole-brain lysate samples gradually increased throughout postnatal development (Figure 1a,c; serum: week 8, *p* < 0.0001; whole-brain: week 8, *p* < 0.0001). At 8 weeks of age, the offspring were systematically treated with the MP5 antibody (100 µg/mouse) via intravenous (i.v.) injection. An isotype-matched IgG was administered to the control mice. ELISA confirmed the effectiveness of this IL-6-blocking treatment, demonstrating a substantial decline in IL-6 levels in serum and brain tissue (Figure 1b,d. STAg +IgG vs. STAg + MP5, serum: *p* < 0.0001; whole-brain: *p* < 0.0001). Behavioral tests commenced one week after treatment to evaluate the enduring effects of STAg-induced MIA and the therapeutic impact of IL-6 inhibition (Figure 2a).

**Figure 3:**
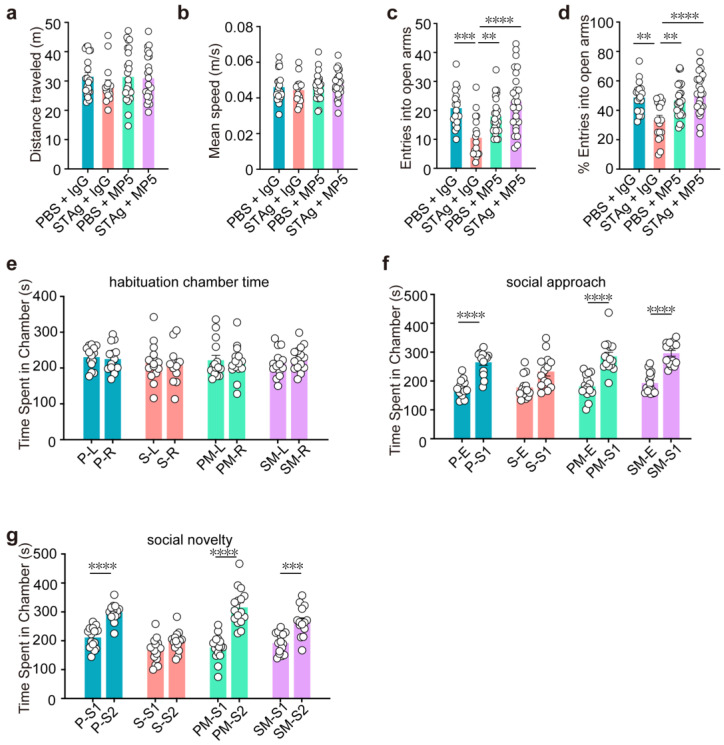
Treatment with IL-6 neutralizing antibody MP5 partially reverses MIA-induced behavioral abnormalities. (**a**,**b**) Open fields test. Distance traveled (**a**) and mean speed (**b**) during a 10-minute open field test (*n* = 20, PBS + IgG, *n* = 19, STAg + IgG; *n* = 20, PBS + MP5; *n* = 19, STAg + MP5; mice from 5 independent dams per group). (**c**,**d**) Elevated plus maze. Entries into open arms (**c**) and percentage of open arm entries (**d**) (*n* = 19, PBS + IgG, *n* = 18, STAg + IgG; *n* = 19, PBS + MP5; *n* = 18, STAg + MP5; mice from 5 independent dams per group). (**e**–**g**) Three-chamber social interaction. (**e**) Time in left/right chambers during habituation (two-way ANOVA with Bonferroni post hoc test). (**f**) Total time spent in the social approach stage in both the empty and stranger mouse 1 (S1) chambers. (**g**) The total amount of time spent in the social novelty stage in the chambers containing stranger mice 1 (S1) and 2 (S2) (*n* = 14, PBS + IgG, *n* = 14, STAg + IgG; *n* = 14, PBS + MP5; *n* = 14, STAg + MP5; mice from 4 independent dams per group). The data are displayed as mean ± s.e.m. Panels **a**–**d**: One-way ANOVA with Bonferroni post hoc test. Panels e–g: Two-way ANOVA with Bonferroni post hoc test. ** *p* < 0.01, *** *p* < 0.001, **** *p* < 0.0001. All statistical information, including sample sizes, group averages, and specific test results, is presented in Extended Data Table S1, and a summary of behavioral outcomes across all tests is provided in Supplementary Table S2.

First, an open field test revealed that the STAg and PBS progeny did not differ in terms of overall distance or speed (Figure 3a,b), suggesting unchanged locomotor activity. While motor coordination deficits are sometimes associated with ASD, in our STAg-induced MIA model, we did not observe significant impairments in general motor coordination, as assessed by a battery of tests, including the rotarod, pole test, beam walking test, and gait analysis. Our focus in this study was on the core autism-like behavioral domains of social interaction, social memory, and repetitive behaviors. Offspring of STAg showed considerably fewer admissions into and time spent in the open field arena’s center (Figure 2c, PBS + IgG vs. STAg + IgG, *p* = 0.0009; Figure 2d, PBS + IgG vs. STAg + IgG, *p* = 0.0008; Figure 2e, PBS + IgG vs. STAg + IgG, *p* = 0.0009), showing that the STAg group had a higher level of anxiety. Notably, inhibition of IL-6 by MP5 antibody treatment increased the locomotor activities of STAg mice at the open field arena’s center, suggesting amelioration of anxiety-like behaviors (Figure 2c–e, STAg + IgG vs. STAg + MP5, *p* < 0.0001). Anxiety-like behaviors in STAg offspring that spent noticeably less time in open arms were further validated by an elevated plus maze test (Figure 2g, PBS + IgG vs. STAg + IgG, *p* = 0.0001; Figure 2h, PBS + IgG vs. STAg + IgG, *p* < 0.0001). Time spent in the open arms was somewhat restored by IL-6 suppression (Figure 2g, STAg +IgG vs. STAg + MP5, *p* = 0.0007; Figure 2h, STAg + IgG vs. STAg + MP5, *p* < 0.0001), demonstrating a rescue of anxiety-like phenotype in these STAg mice. Consistently, both the number of entries into open arms and the percentage of entries into open arms showed similar trends (Figure 3c, PBS + IgG vs. STAg + IgG, *p* = 0.0008; STAg + IgG vs. STAg + MP5, *p* < 0.0001; Figure 3d, PBS + IgG vs. STAg + IgG, *p* = 0.0019, STAg +IgG vs. STAg + MP5, *p* < 0.0001).

To assess social behaviors, we looked at reciprocal social connections. During the habituation phase, chamber time showed no difference between PBS and STAg offspring in time spent in the left versus right chamber (Figure 3e). In the social approach and social novelty tests, PBS offspring showed a preference for the stranger-containing chamber, whereas STAg offspring did not; treatment with IL-6-neutralizing antibody MP5 restored this preference (Figure 3f, PBS + IgG S1 vs. E, PBS + MP5 S1 vs. E, STAg + MP5 S1 vs. E, *p* < 0.0001. Figure 3g, PBS + IgG S2 vs. S1, PBS + MP5 S2 vs. S1, *p* < 0.0001, STAg + MP5 S2 vs. S1, *p* = 0.0005).

**Figure 4:**
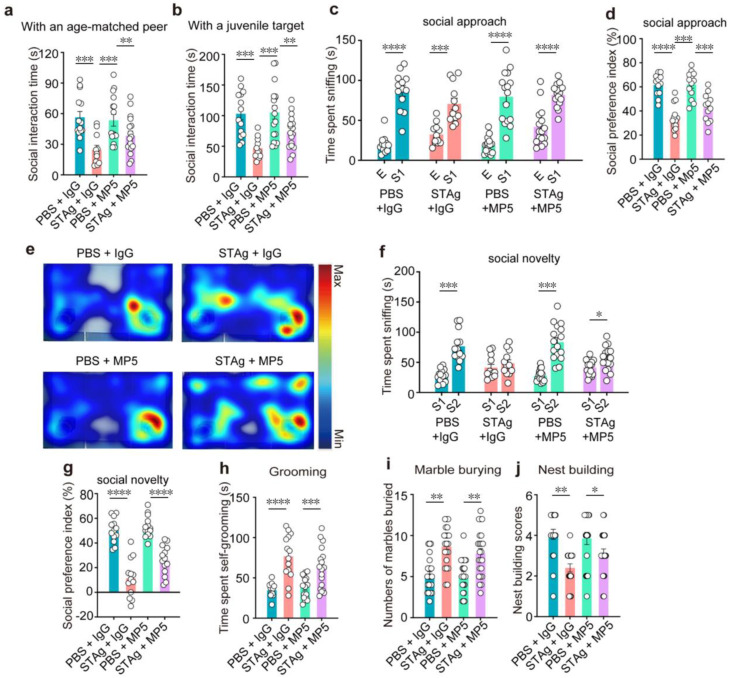
The IL-6-neutralizing antibody MP5 does not improve MIA-induced social preference deficits and repetitive behaviors. (**a**) Reciprocal social interactions. Active social interactions with an age-matched peer (**a**) or a juvenile target (**b**) measured after injection of IgG or MP5 in PBS or STAg-MIA offspring (*n* = 14, PBS + IgG, *n* = 13, STAg + IgG; *n* = 14, PBS + MP5; *n* = 13, STAg + MP5; mice from 4 independent dams per group). (**c**–**g**) Three-chamber social interaction test. (**e**) Heatmap illustrating how much time was spent in the three-chamber arena throughout the stage of social novelty. (**c**) The total amount of time spent sniffing the cage with stranger mouse 1 and the cage that was empty. (**d**) The social preference index was analyzed in the social approach stage. (**f**) The total amount of time spent sniffing stranger mouse 1 and stranger mouse 2. During the social novelty stage, the social preference index (**g**) was calculated (*n* = 14, PBS + IgG, *n* = 14, STAg + IgG; *n* = 14, PBS + MP5; *n* = 14, STAg + MP5; mice from 4 independent dams per group). (**h**) Self-grooming duration from PBS and STAg adult offspring injected with IgG or MP5 (*n* = 15, PBS + IgG, *n* = 15, STAg + IgG; *n* = 15, PBS + MP5; *n* = 15, STAg + MP5; mice from 5 independent dams per group). (**i**) The marble-burying test. The quantity of buried marbles for four groups of mice in 30 min (*n* = 20, PBS + IgG, *n* = 20, STAg + IgG; *n* = 20, PBS + MP5; *n* = 20, STAg + MP5; mice from 5 independent dams per group). (**j**) Test for nesting. Nesting scores were assessed in PBS and STAg adult offspring following treatment with IgG or MP5 (*n* = 19, PBS + IgG, *n* = 19, STAg + IgG; *n* = 19, PBS + MP5; *n* = 19, STAg + MP5; mice from 5 independent dams per group). The data are shown as mean ± s.e.m. Panels (**a**,**b**,**d**,**g**–**j**): One-way ANOVA with Bonferroni post hoc test. Panels (**c**,**f**): Two-way ANOVA with Bonferroni post hoc test. * *p* < 0.05, ** *p* < 0.01, *** *p* < 0.001, **** *p* < 0.0001. All statistical information, including sample sizes, group averages, and specific test results, is presented in Extended Data Table S1, and a summary of behavioral outcomes across all tests is provided in Supplementary Table S2.

Although chamber preference was rescued by MP5, direct social interaction remained impaired. STAg offspring interacted with an age-matched peer (Figure 4a, PBS + IgG vs. STAg + IgG, *p* = 0.0003) and a juvenile target mouse (Figure 4b, PBS + IgG vs. STAg + IgG, *p* = 0.0008) for a much shorter period of time than the PBS group. Administration of MP5 antibody against IL-6 did not increase social interaction time with stranger mice (Figure 4a, PBS + MP5 vs. STAg + MP5, *p* = 0.0028; 4b, PBS + MP5 vs. STAg + MP5, *p* = 0.0012). Next, in the three-chamber social interaction (SI) tests, sociability (stage 2) and social recognition memory (stage 3) were assessed. At stage 2, both STAg and PBS offspring mice spent more time sniffing stranger 1 mice (S1) over the empty cage (E) (Figure 4c, PBS + IgG S1 vs. E, *p* < 0.0001; STAg + IgG S1 vs. E, *p* = 0.0003; PBS + MP5 S1 vs. E, *p* < 0.0001; STAg + MP5 S1 vs. E, *p* < 0.0001), but the social preference index was significantly lower in the STAg group (Figure 4d, PBS + IgG vs. STAg + IgG, *p* < 0.0001), indicating impaired sociability in these mice. IL-6 inhibition by MP5 antibody did not restore the social preference index in the STAg offspring (Figure 4d, PBS + MP5 vs. STAg + MP5, *p* = 0.0002). In the social novelty test at stage 3, PBS offspring spent a notably longer period of time sniffing stranger 2 mice (Figure 4f, PBS + IgG S2 vs. S1, *p* = 0.0004; PBS + MP5 S2 vs. S1, *p* = 0.0003; STAg + MP5 S2 vs. S1, *p* = 0.0446), resulting in a higher social preference index (Figure 4g, PBS + IgG vs. STAg + IgG, *p* < 0.0001; PBS + MP5 vs. STAg + MP5, *p* < 0.0001). IL-6 inhibition failed to increase the time sniffing stranger 2 over stranger 1 mice or to restore the social preference index in the STAg offspring (Figure 4e–g).

The main indicators of an ASD diagnosis are repetitive, stereotypical behaviors, as well as deficiencies in social interaction and communication. We then conducted grooming tests and marble burying tests to assess repetitive behaviors. In comparison to the PBS group, the STAg group groomed for around twice as long (Figure 4h, PBS + IgG vs. STAg + IgG, *p* < 0.0001; PBS + MP5 vs. STAg + MP5, *p* = 0.0009). In the marble burial test, the STAg offspring buried about eight marbles in 30 min, compared to the PBS group’s average of about five (Figure 4i, PBS + IgG vs. STAg + IgG, *p* = 0.0013; PBS + MP5 vs. STAg + MP5, *p* = 0.0015). IL-6 inhibition by the MP5 antibody did not rescue these behavioral phenotypes (Figure 4h,i). Additionally, unlearned nest-building performance, indicating a health condition, was not affected in the STAg group by IL-6 inhibition in the STAg group (Figure 4j, PBS + IgG vs. STAg + IgG, *p* = 0.0098; PBS + MP5 vs. STAg + MP5, *p* = 0.0380).

Collectively, our findings show that adult offspring with prenatal exposure to STAg-elicited MIA have characteristics similar to those with anxiety and ASD. Systematic inhibition of IL-6 signaling by antibody-mediated neutralization ameliorates anxiety and social interaction deficits in this mouse model, but did not rescue impairments in social recognition, social novelty, or repetitive behaviors.

## 4. Discussion

Our study reveals that prenatal exposure to STAg-induced MIA triggers autism-like behaviors and anxiety in adult offspring mice, emphasizing the pivotal influence of MIA on neurodevelopmental outcomes [7]. This finding is in line with other research showing that immunological stimulation during crucial stages of fetal brain development causes long-lasting alterations in synapse structure, disturbances in neurotransmitter homeostasis, and persistent microglia activation. These neurodevelopmental abnormalities ultimately result in behavioral phenotypes such as autism-like behavioral abnormalities and increased anxiety in offspring [31,32,33]. Notably, systemic inhibition of IL-6 signaling using the MP5 antibody alleviated anxiety-like behaviors, including self-grooming, and deficits in social interaction, highlighting an important role of IL-6 in these behavioral alterations. IL-6 has been widely recognized as a key inflammatory mediator in the MIA model. Studies on mice have confirmed that it is linked to social behavior and anxiety in offspring [34,35]. Additionally, human epidemiological data have shown a strong association between poor fetal brain development and elevated IL-6 levels during pregnancy, as well as an increased risk of neuropsychiatric problems in offspring [36,37]. The three-chamber social preference test is a robust measure of the initial social approach and investigation drive. A positive result in this test (stage 2) reflects an improvement in the foundational motivation for social contact. In contrast, the direct interaction test is a more demanding assay of active and engaged social behavior. The lack of effect in this latter test indicates that while motivational deficits were alleviated, higher-order social cognitive or executive functions likely remained impaired. Therefore, these findings should not be viewed as conflicting, but rather as highlighting an important nuance: our intervention appears to selectively target the motivational component of sociability. In contrast to the therapeutic effects observed with adoptive Treg transfer therapy in STAg offspring [7], antibody-mediated inhibition of IL-6 did not improve repetitive behaviors or social recognition impairments, indicating that these traits may arise from separate pathways or that IL-6 plays a less dominant role in their etiology. These findings imply that several inflammatory factor processes may be responsible for the behavioral abnormalities associated with ASD.

A technical limitation of this study is that since most behavioral tests used to assess autism-like phenotypes in mouse models require mice to be at an adult age (typically 8 weeks or older) for valid and reliable performance, IL-6 inhibition and the evaluation of its effects were conducted in adult mice. Therefore, we cannot rule out possible therapeutic effects if IL-6 inhibition is applied in neonatal mice. Secondly, antibodies can effectively neutralize IL-6 in the peripheral circulation system, but may not exert significant effects in the brain parenchyma, due to the blood–brain barrier (BBB). Moreover, although our prior work showed that IL-6 is elevated in multiple brain regions and predominantly expressed by astrocytes in MIA offspring [7], the present study did not perform brain region-specific or downstream pathway analyses, leaving the mechanistic link between IL-6 signaling and behavioral outcomes incompletely defined. A further limitation is that only male offspring were tested, which introduces a sex bias. While this choice aligns with the well-established male-biased MIA model, it does not allow assessment of IL-6 inhibition effects in females. Future studies including both sexes will be needed to determine whether these findings generalize and to explore potential sex differences in IL-6–mediated neuroimmune mechanisms. Finally, potential variations in maternal behavior, which were not examined in this study, could influence offspring behavioral outcomes independently of MIA. 

We propose that the systemic anti-inflammatory effects of IL-6 neutralization may indirectly ameliorate the inflammatory conditions within the brain, likely through the modulation of other circulating cytokines and inflammatory mediators. Our prior publication has shown that IL-6 is elevated in the brains of MIA offspring and contributes to the neuroinflammatory state, and demonstrated that the adoptive transfer of Tregs effectively reversed most autism-like phenotypes [7]. This was attributed to Tregs’ broad and potent immunosuppressive functions, which extend beyond a single cytokine pathway. In contrast, the current study specifically targeted IL-6 in isolation. The finding that IL-6 inhibition alone failed to rescue core ASD-like phenotypes underscores the limitations of single-cytokine interventions and supports our previous conclusion: Comprehensive immunomodulatory strategies—such as those exerted by Tregs—are likely required to address the complex and multifaceted behavioral and neuroinflammatory consequences of MIA. These findings shed light on the intricate relationship between immune dysregulation and ASD-related phenotypes, suggesting that while therapies targeting IL-6 may offer a viable strategy for mitigating a subset of behavioral challenges in ASD, broader therapeutic approaches may be necessary to manage the full spectrum of symptoms.

## Data Availability

The data presented in this study are available on request from the corresponding author due to reasonable request.

## Supplementary Materials

The following supporting information can be downloaded at: https://www.mdpi.com/article/10.3390/cimb47100852/s1.

## Abbreviations

The following abbreviations are used in this manuscript:

MIA	Maternal immune activation
ASD	Autism spectrum disorder
STAg	Soluble tachyzoite antigen from *Toxoplasma gondii*
MP5	Monoclonal antibody MP5-20F3
